# PrfA regulation offsets the cost of *L* *isteria* virulence outside the host

**DOI:** 10.1111/1462-2920.12980

**Published:** 2015-08-27

**Authors:** Radhakrishnan B. Vasanthakrishnan, Aitor de las Heras, Mariela Scortti, Caroline Deshayes, Nick Colegrave, José A. Vázquez‐Boland

**Affiliations:** ^1^ Microbial Pathogenesis GroupSchool of Biomedical SciencesUniversity of EdinburghEdinburghUK; ^2^ School of Biological SciencesUniversity of EdinburghEdinburghUK; ^3^ The Roslin InstituteUniversity of EdinburghEdinburghUK; ^4^ Centre for ImmunityInfection & EvolutionUniversity of EdinburghEdinburghUK; ^5^ Grupo de Patogenómica BacterianaUniversidad de LeónLeónSpain

## Abstract

Virulence traits are essential for pathogen fitness, but whether they affect microbial performance in the environment, where they are not needed, remains experimentally unconfirmed. We investigated this question with the facultative pathogen *L* *isteria monocytogenes* and its PrfA virulence regulon. PrfA‐regulated genes are activated intracellularly (PrfA ‘ON’) but shut down outside the host (PrfA ‘OFF’). Using a mutant PrfA regulator locked ON (PrfA*) and thus causing PrfA‐controlled genes to be constitutively activated, we show that virulence gene expression significantly impairs the listerial growth rate (μ) and maximum growth (A) in rich medium. Deletion analysis of the PrfA regulon and complementation of a *L. monocytogenes* mutant lacking all PrfA‐regulated genes with PrfA* indicated that the growth reduction was specifically due to the unneeded virulence determinants and not to pleiotropic regulatory effects of PrfA ON. No PrfA*‐associated fitness disadvantage was observed in infected eukaryotic cells, where PrfA‐regulated virulence gene expression is critical for survival. Microcosm experiments demonstrated that the constitutively virulent state strongly impaired *L* *. monocytogenes* performance in soil, the natural habitat of these bacteria. Our findings provide empirical proof that virulence carries a significant cost to the pathogen. They also experimentally substantiate the assumed, although not proven, key role of virulence gene regulation systems in suppressing the cost of bacterial virulence outside the host.

## Introduction

The ability of a microbe to infect and cause harm (virulence) correlates with its multiplication rate within the host, itself a direct determinant of between‐host transmission success (Read, 1994; Lipsitch and Moxon, 1997). High virulence, however, may immobilize or cause the death of the host, impairing transmission to new hosts and hence pathogen fitness. Virulence has thus been theorized to hinge on a trade‐off balance with transmissibility and to be potentially costly to the pathogen (Anderson and May, 1981; Antia *et al*., 1994; Bull, 1994; Alizon *et al*., 2009). This relationship is easily intuited for microparasites depending on a live host for transmission (i.e. obligate pathogens) and is at the core of virulence theory (Bull and Lauring, 2014). However, whether microbial virulence also affects the performance of indirectly transmitted pathogens in the environment remains to be clarified and is largely neglected by evolutionary models.

Virulence determinants have specifically evolved to confer an advantage within the host, and the gratuitous expression of microbial traits in a situation in which they are not required is known to carry fitness penalties (Nguyen *et al*., 1989; Eames and Kortemme, 2012). Despite the obvious potential significance for pathogen evolution, experimental information about the costs associated with unneeded virulence traits in a non‐host system is essentially lacking. A number of studies with phytopathogens have examined the fitness costs of ‘avirulence’ gene mutations to virulence in susceptible plant populations without the matching resistance (R) gene (where the pathogen's avirulence/virulence gene is irrelevant) (Leach *et al*., 2001; Bahri *et al*., 2009; Huang *et al*., 2010; Montarry *et al*., 2010). These studies have generally measured the cost of virulence via the effects on within‐host fitness attributes (e.g. *in planta* multiplication, amount of disease symptoms or pathogen released from leaves) but not on saprophytic growth and survival (Sacristan and Garcia‐Arenal, 2008). In animal pathogens, a recent report on *Salmonella* addressed the cost of virulence factors in *in vitro* culture (Sturm *et al*., 2011). In this study, Sturm and colleagues showed that expression of the type III secretion system (TTSS)‐1 was associated with significant growth retardation. Gene deletion analysis suggested that the growth defect was at least in part attributable to TTSS‐1 virulence factor expression, although the possibility that it was also due to global, pleiotropic regulatory effects was not excluded (Sturm *et al*., 2011).

**Figure 1:**
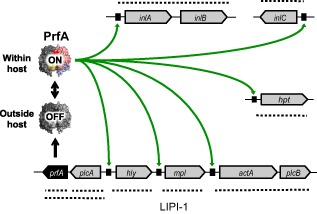
Schematic of *L* *. monocytogenes* PrfA virulence regulon and ON–OFF PrfA switching. Dotted lines indicate relevant transcriptional units.

*Listeria monocytogenes* is a prototypic facultative pathogen that can live both as a soil saprotroph or an intracellular parasite of animals and people (Vazquez‐Boland *et al*., 2001b; Freitag *et al*., 2009). Listerial virulence is conferred by a set of proteins that promote host cell invasion (internalins InlA and InlB), phagocytic vacuole escape (pore‐forming toxin Hly, phospholipases PlcA and PlcB, metalloprotease Mpl), cytosolic replication (sugar phosphate transporter Hpt) and actin‐based cell‐to‐cell spread (surface protein ActA, internalin InlC) (Cossart, 2011). The genes encoding these nine virulence factors are coordinately regulated by the transcriptional activator PrfA (Mengaud *et al*., 1991; Chakraborty *et al*., 1992) (Fig. 1). PrfA‐regulated genes are normally very weakly expressed outside the host but strongly induced during intracellular infection (Moors *et al*., 1999; Shetron‐Rama *et al*., 2002; Chatterjee *et al*., 2006; Joseph *et al*., 2006; Toledo‐Arana *et al*., 2009). This activation is thought to require PrfA to allosterically switch from its native, weakly active (‘OFF’) conformation to a highly active (‘ON’) state (Scortti *et al*., 2007; de las Heras *et al*., 2011) and is essential for *Listeria* virulence (Deshayes *et al*., 2012). Single amino acid substitutions that lock PrfA in an ‘always‐ON’ (PrfA*) state have been identified (Ripio *et al*., 1997; Vega *et al*., 2004; Wong and Freitag, 2004). *Listeria monocytogenes* mutants carrying one such PrfA* substitution, G145S, constitutively express the PrfA‐regulated genes *in vitro* to levels similar to the wild type during intracellular infection (Ripio *et al*., 1997; Vega *et al*., 2004; Deshayes *et al*., 2012). prfA*^G145S^ mutants therefore provide a unique tool to investigate the cost of virulence traits in non‐host conditions.

Taking advantage of the properties conferred by the prfA* allele, we show that virulence gene activation imposes a significant burden on *L. monocytogenes* outside the host. We also show that this burden limits the survival and competitive ability of *L. monocytogenes* in soil. Our data provide the first formal demonstration that the virulence traits that make a microbe pathogenic entail a significant fitness cost. We also experimentally substantiate that a primary key role of virulence gene regulation systems in facultative pathogens is to neutralize the cost of virulence outside the host, thereby maximizing between‐host pathogen fitness in the environmental reservoir.

## Results

When first identified in our laboratory (Ripio *et al*., 1996; 1997), we observed that prfA* mutants exhibited impaired growth in broth medium, suggesting a fitness defect (unpubl. data). The prfA*‐associated growth reduction was also noted by others, although the effect was relatively minor compared with wild‐type *prfA* (*prfA* ^WT^) and was not statistically confirmed (Marr *et al*., 2006). More recently, *L. monocytogenes* bacteria carrying prfA* alleles were found to have increased sensitivity to stress and a competitive disadvantage upon repeated passage in broth culture (Bruno and Freitag, 2010), although no growth defect in rich medium was directly observed in monoculture (Port and Freitag, 2007; Bruno and Freitag, 2010). The interpretation of these reports was complicated by possible regulatory interference of PrfA ON with listerial carbon nutrition/metabolism (Marr *et al*., 2006; Bruno and Freitag, 2010). Moreover, effects on fitness could have been obscured in these studies by the use of strains *trans*‐complemented with the *prfA* gene on a multicopy plasmid (Marr *et al*., 2006), or carrying enzymatic and antibiotic resistance cassettes under the control of a PrfA‐dependent promoter (Port and Freitag, 2007; Bruno and Freitag, 2010).

### Cost of PrfA activation *in vitro*

To avoid possible confounding effects due to the potential burden introduced by multicopy plasmids or reporter genes, we investigated the fitness consequences of PrfA regulon activation using a naturally occurring prfA*^G145S^ strain (P14A) (Ripio *et al*., 1997) and an isogenic, unmarked *prfA* ^WT^ allelic exchange revertant thereof (P14^Rev^). The latter was obtained by double homologous recombination using fosfomycin to counterselect the original prfA* genotype (see *Experimental procedures*). This selection strategy is based on the ability of the listerial PrfA‐dependent sugar phosphate permease Hpt to confer susceptibility to fosfomycin when the PrfA system is activated (Scortti *et al*., 2006). Bacterial fitness was measured by determining the exponential growth rate (μ) and maximum growth yield (A) in brain–heart infusion (BHI) broth, a rich culture medium in which *Listeria* growth is optimal and wild‐type PrfA‐dependent gene expression is maximally downregulated at 37°C (Ripio *et al*., 1996; 1997; Shetron‐Rama *et al*., 2003). As controls, an isogenic in‐frame *prfA* deletant (Δ*prfA*) and the parent *prfA* ^WT^ strain of P14A (isolate P14) were also tested.

**Figure 2:**
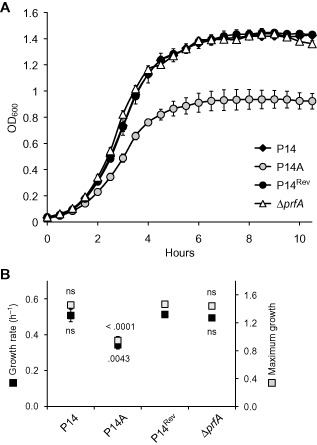
Growth in BHI of *L* *. monocytogenes* P14A (*prf* A*), isogenic P14^Rev^ (*prf* *A* ^WT^ allele replacement revertant) and Δ*prf* *A* derivatives of P14A, and the wild‐type parent strain P14. Mean ± SEM of four experiments. A. Growth curves. B. Growth rate (μ) and maximum growth (A) expressed in OD_600_ units. P14^Rev^ was used as the reference in post‐hoc multiple comparisons. Numbers indicate *P* values; ns, not significant.

P14A exhibited a clear growth defect in BHI, as evidenced by its significantly lower μ and A values (F_3,10_ = 8.07 *P* = .005 and 54.98 *P* < .0001 respectively) (Fig. 2). Replacement of P14A's prfA* allele by *prfA* ^WT^ (P14^Rev^) restored growth to wild‐type (P14) levels. On the other hand, the growth dynamics of P14 and P14^Rev^, both expressing a PrfA^WT^ protein, was identical to that of the Δ*prfA* strain lacking PrfA (Fig. 2). These data indicate (i) that the constitutively active PrfA*^G145S^ protein, driving high (‘*in vivo*’ equivalent) levels of PrfA‐dependent gene expression in *in vitro* conditions (Ripio *et al*., 1997; Deshayes *et al*., 2012), significantly impairs *L. monocytogenes* fitness in rich medium; and (ii) that PrfA^WT^, associated with negligible levels of PrfA‐dependent gene expression *in vitro* (Ripio *et al*., 1997; Deshayes *et al*., 2012), has a neutral effect on *L. monocytogenes* performance.

### PrfA* does not impair *L* *. monocytogenes* fitness in infected host cells

Since PrfA‐regulated virulence determinants are unlikely to be necessary for extracellular growth *in vitro*, the fitness disadvantage observed with the prfA* allele in BHI could reflect the burden typically associated with expressing dispensable gene products (Dong *et al*., 1995; Stoebel *et al*., 2008; Shachrai *et al*., 2010). If this explanation is correct, then no significant growth impairment is expected to occur in an infection setting, where bacterial fitness depends on the expression of virulence genes. To confirm this, we compared the behaviour of the prfA* and *prfA* ^WT^ bacteria in intracellular proliferation assays in eukaryotic cell monolayers.

**Figure 3:**
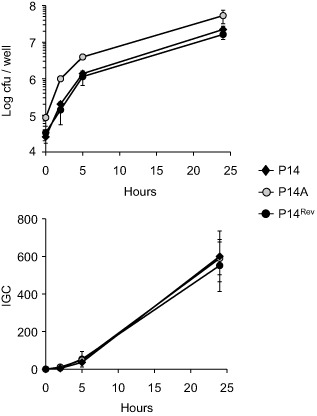
Intracellular proliferation of *L* *. monocytogenes prf* A* (strain P14A) and *prf* *A* ^WT^ (P14A isogenic wild‐type allele‐replacement revertant P14^Rev^ and parent strain P14) in human HeLa cells. Upper panel, intracellular colony forming units (cfu); lower panel, data expressed as normalized intracellular growth coefficient (IGC, see *Experimental procedures*). Mean ± SEM of three experiments.

P14A did not differ from P14^Rev^ (and P14) in intracellular growth in HeLa cells (F_2,3_ = 0.04 *P* = .9575) (Fig. 3). This result is in agreement with previous data showing that prfA* and *prfA* ^WT^ *L. monocytogenes* have similar or comparable virulence *in vivo* in mice and in infected cells (Ripio *et al*., 1996; Shetron‐Rama *et al*., 2003; Bruno and Freitag, 2010; Deshayes *et al*., 2012). Thus, despite the significant growth defect observed *in vitro* in rich medium, the PrfA* protein did not seem to impair *L. monocytogenes* fitness *in vivo* in a host system. This is consistent with the notion that PrfA* is locked in the ON state presumably adopted by PrfA^WT^ *in vivo* during infection, resulting in similar levels of virulence gene expression for both prfA* and *prfA* ^WT^ bacteria within host cells (de las Heras *et al*., 2011; Deshayes *et al*., 2012).

### The fitness cost is due to PrfA regulon components

**Figure 4:**
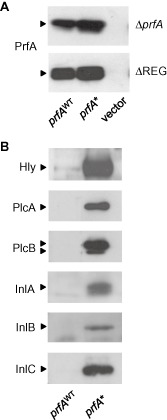
Western immunoblot analysis. A. Detection of PrfA in cell extracts of Δ*prf* *A* and ΔREG bacteria complemented with *prf* *A* ^WT^ or *prf* A* alleles. Protein loaded: 10 μg. B. Detection of selected PrfA‐dependent virulence factors in the cell extracts or culture supernatants of Δ*prf* *A* complemented with *prf* *A* ^WT^ or *prf* A* alleles. The two arrows in PlcB indicate the unprocessed and mature form of the enzyme. Protein loaded per lane: 20 μg, 5 μg for Hly.

The growth reduction associated with the prfA* allele in nutrient‐rich BHI could be due to the cost of expressing unneeded virulence products, or alternatively to PrfA ON interfering with some listerial housekeeping function important for listerial growth, as previously suggested (Marr *et al*., 2006). To address this question, we constructed a P14A mutant lacking the entire PrfA regulon (ΔREG), i.e. *Listeria* pathogenicity island 1 encompassing the *prfA*, *plcA*, *hly*, *mpl*, *actA* and *plcB* genes (LIPI‐1), the internalin loci *inlAB* and *inlC*, and the organophosphate transporter gene *hpt* (also known as *uhpT*) (Fig. 1). ΔREG was complemented with either *prfA* ^WT^ (from P14) or prfA*^G145S^ (from P14A) inserted in monocopy in a permissive site of the listerial chromosome using an integrative vector (pPL2) (Lauer *et al*., 2002; Deshayes *et al*., 2012). P14A Δ*prfA*, which possesses the entire PrfA regulon except the deleted *prfA* gene, was also complemented with the same *prfA* constructs as a control. Western blot analyses confirmed that the PrfA protein was correctly expressed in *prfA*‐complemented ΔREG and Δ*prfA* (Fig. 4A). They also confirmed that the prfA* and *prfA* ^WT^ constructs induced, respectively, the expected high and low/undetectable expression levels of PrfA‐regulated products in BHI (Fig. 4B).

**Figure 5:**
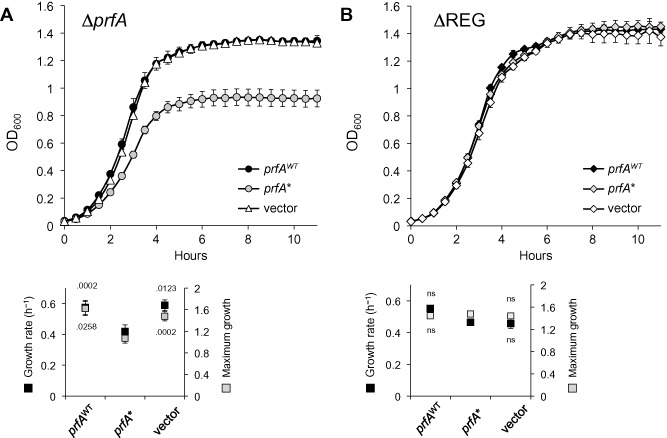
Growth in BHI of (A) Δ*prf* *A* and (B) ΔREG, each complemented with *prf* *A* ^WT^, *prf* A* or empty vector. Below, corresponding μ (growth rate) and A (maximum growth) values expressed in OD_600_ units; *prf* A*‐complemented bacteria were used as the reference in post‐hoc multiple comparisons. Mean ± SEM of at least three experiments. Numbers indicate *P* values; ns, not significant. The Δ*prf* *A* and ΔREG growth curves, shown separately for clarity, were determined in the same set of experiments.

Complementation of Δ*prfA* with the prfA* allele, but not *prfA* ^WT^ or empty vector, caused growth inhibition, with significant reduction in both μ and A (F_2,8_ = 8.17 *P* = .0117 and 34.04 *P* < .0001 respectively) (Fig. 5A). This mirrored the previous data with the isogenic strains carrying the *prfA* gene in its native chromosomal location, confirming that the growth reduction was solely due to the activity of PrfA*. In contrast, no significant differences were observed between the complemented ΔREG strains (μ *P* = .1397, A *P* = .9142) (Fig. 5B), or between these and Δ*prfA* complemented with *prfA* ^WT^ or empty vector (μ *P* = .4104, A *P* = .1719). These data show that the growth reduction caused by PrfA ON requires the presence of the PrfA‐dependent virulence genes on the listerial chromosome.

**Figure 6:**
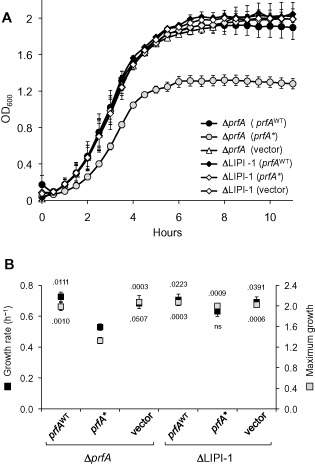
Growth in BHI of ΔLIPI‐1 complemented with *prf* *A* ^WT^, *prf* A* or empty vector. Δ*prf* *A* bacteria complemented with *prf* *A* ^WT^, *prf* A* or empty vector were used as a control. A. Growth curves. B. Corresponding μ (growth rate) and A (maximum growth) values expressed in OD_600_ units. Mean ± SEM of three experiments. Δ*prf* *A* complemented with *prf* A* used as reference in post‐hoc multiple comparison. Numbers indicate *P* values; ns, not significant.

Partial PrfA regulon mutants in P14A were analysed to determine the contribution of specific PrfA‐regulated loci to the fitness loss. Deletion of the internalin genes *inlAB* and *inlC* or the *hpt* monocistron did not relieve the growth defect caused by PrfA* (Fig. S1). In contrast, deletion of LIPI‐1 rescued the growth defect in the presence of prfA* (Fig. 6). Some recovery of the wild‐type phenotype was observed for single *hly* or *actA* deletion mutants within LIPI‐1, although the effect was not statistically significant (Figs S2 and S3). Thus, the PrfA*‐associated growth impairment is mainly attributable to LIPI‐1 and depends on the expression of several PrfA‐regulated genes. Together, our results are consistent with the growth reduction caused by PrfA ON being due to the burden associated with the expression of PrfA regulon virulence determinants.

### PrfA switch‐off is required for optimal fitness in soil

We next sought to investigate the effect of PrfA activation on fitness in a non‐host model more closely approximating the conditions encountered by *L. monocytogenes* in nature. Soil rich in decaying plant matter is considered to be the main *Listeria* environmental reservoir (Weis and Seeliger, 1975; Vazquez‐Boland *et al*., 2001b; Freitag *et al*., 2009; Vivant *et al*., 2013) and was chosen for these experiments. Sterile topsoil of neutral pH was used to ensure optimal *L. monocytogenes* growth/survival (Botzler *et al*., 1974; McLaughlin *et al*., 2011; Locatelli *et al*., 2013; Vivant *et al*., 2013). P14A (prfA*) and its isogenic P14^Rev^ (*prfA* ^WT^) and Δ*prfA* derivatives were inoculated in axenic microcosms at a dose of ≈ 6 × 10^6^ cfu g^−1^, and viable bacterial numbers in soil were regularly monitored for 17 days by plate counting. Although the pPL2 vector had previously demonstrated stable chromosomal integration in a variety of conditions (*in vitro* in culture media or *in vivo* in infected cells and mice) (Lauer *et al*., 2002; Deshayes *et al*., 2012; this study), the *prfA* ^WT^ and prfA* pPL2 constructs (and control empty vector) were rapidly lost in soil by the complemented Δ*prfA* strain (within the first 48 h) and could not be used.

**Figure 7:**
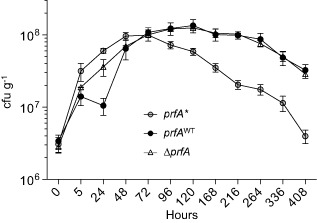
Monoculture experiments in soil. Microcosms were seeded with ≈ 6 × 10^6^ cfu g^−1^ of *L* *. monocytogenes prf* A* (P14A), *prf* *A* ^WT^ (P14^Rev^) or Δ*prf* *A*, and the bacterial population dynamics for each strain regularly monitored in soil by plate counting during static incubation at room temperature. See *Experimental procedures* for details. Results expressed as mean cfu g^−^1 ± SEM of three replicates. The *prf* A* and *prf* *A* ^WT^ alleles remained stable throughout the experiments (see Fig. S5).

P14A again showed significantly different behaviour (genotype × time points F_22,72_ = 5.02 *P* < .0001; two‐way analysis of variance (ANOVA) with Tukey's post‐hoc multiple comparisons): after an initial population increase for the three strains, P14A counts steadily dropped from day 3, while P14^Rev^ and Δ*prfA* continued to grow until day 5, followed by stabilization until declining after day 11 (Fig. 7). Thus, consistent with our observations in rich medium, prfA* bacteria also exhibited diminished fitness in soil compared with *prfA* ^WT^ and Δ*prfA* bacteria.

### Competition experiments

To internally control for possible inter‐sample variation in growth due to physicochemical/nutritional microenvironment heterogeneity in soil (Vivant *et al*., 2013), the strains were tested in mixed culture in the same soil microcosms. This approach also permits direct determination of the competitive ability and an estimate of the strength of selection acting against the less fit genotype (Lenski, 1992). Either prfA* or *prfA* ^WT^ bacteria were co‐inoculated in a ≈ 1:1 ratio with Δ*prfA* used as a common reference. This allowed confirmation of the relative frequencies of the competing genotypes by polymerase chain reaction (PCR) screening of the specific deletion in Δ*prfA* (see *Experimental procedures*).

**Figure 8:**
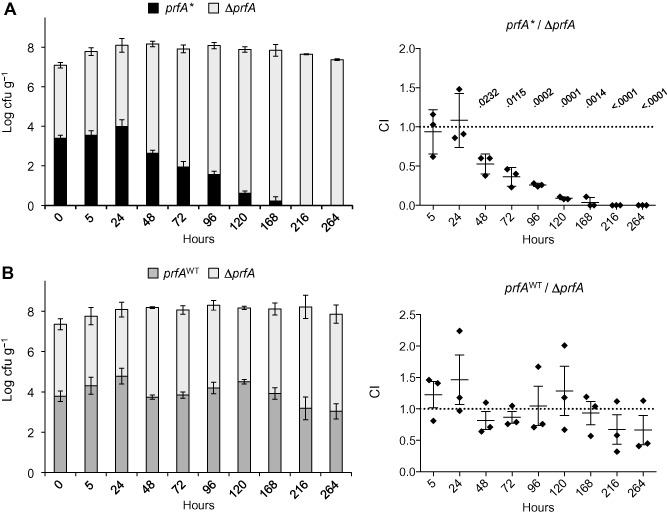
Competition experiments in soil. (A) *prf* A* (P14A) versus Δ*prf* *A*. (B) *prf* *A* ^WT^ (P14^Rev^) versus Δ*prf* *A*. Microcosms were inoculated with ≈ 10^7^ cfu g^−1^ of 1:1 mixes of the indicated *L. monocytogenes* strains. Left panels, bar charts: bar height indicates log total cfu g^−1^; black and grey areas within bars indicate the proportion of competing bacteria. Right panels, competitive index (CI). *P* values for statistically significant differences with the reference value 1 are indicated (see *Experimental procedures*). Mean ±SEM of three replicates.

prfA* bacteria were clearly outcompeted by Δ*prfA* after the first 24 h [competitive index (CI) < 1] until their total disappearance by day 9 (Fig. 8A). In contrast, no differences in the relative fitness of *prfA* ^WT^ and Δ*prfA* genotypes (CI not significantly different from 1) were observed throughout the experiment (Fig. 8B). These data indicate that (i) the burden imposed by the activation of the PrfA virulence regulon compromises *L. monocytogenes* survival in soil, and (ii) the virulence‐associated fitness cost in soil is effectively compensated by the ON–OFF switchable PrfA regulator.

## Discussion

Microbial growth is a correlate of the fitness status of the prokaryotic cell and responds to the principle of cost–benefit optimality. To ensure maximal fitness, microbial cells need to optimize the allocation of limited resources to competing traits (Dekel and Alon, 2005; Molenaar *et al*., 2009; Berkhout *et al*., 2013). This is often achieved by coupling gene expression to beneficial processes under specific conditions, as classically illustrated by studies with the *lac* operon or antibiotic resistance determinants (Koch, 1983; Nguyen *et al*., 1989; Dekel and Alon, 2005; Stoebel *et al*., 2008; Eames and Kortemme, 2012). Here we analysed the fitness consequences of expressing virulence traits in conditions in which they are not directly beneficial, i.e. during saprophytic growth outside the host. Notwithstanding its undeniable potential significance in pathogen evolution and transmission dynamics, this question had been insufficiently investigated. Using *L. monocytogenes* and a mutant form of its master virulence regulator, PrfA*^G145S^ (Ripio *et al*., 1997), which causes virulence genes to be constitutively expressed *in vitro* to the same high levels seen *in vivo* during infection (de las Heras *et al*., 2011; Deshayes *et al*., 2012), we demonstrate that virulence traits impose a significant burden on bacterial fitness. The fitness disadvantage was evident in extracellular conditions but not in infected cells where the virulence products are indispensable, reflecting that, during infection, the burden associated with virulence factor synthesis is compensated by the beneficial effects on within‐host fitness. Using a soil model, we further show, for the first time, that the virulence‐associated fitness cost translates into significantly impaired bacterial survival in an environmental milieu relevant for pathogen transmission.

PrfA* had no effect on growth in the absence of the target PrfA regulon genes, indicating that the impaired performance was clearly linked to the expression of the virulence factors and not due to PrfA ON disturbing an unrelated housekeeping or metabolic pathway(s). A possible explanation is that some PrfA regulon product(s) might exert a direct inhibitory effect on *L. monocytogenes* via unknown mechanisms. Alternatively, and more plausibly, the PrfA*‐associated growth deficiency may be the consequence of the gratuitous expression of unneeded PrfA regulon products. Indeed, growth reduction is the typical penalty response observed when wasteful proteins are expressed by bacterial cells, aka protein cost (Dong *et al*., 1995; Dekel and Alon, 2005; Stoebel *et al*., 2008; Shachrai *et al*., 2010). The growth deficiency was readily apparent in monoculture in resource‐replete conditions, indicating that the impact of PrfA regulon activation on *Listeria* fitness is substantial. LIPI‐1, which contains six of the nine PrfA‐regulated genes (Fig. 1), appeared to account for the entire burden. Growth rate (μ) and growth yield (A) were both impaired, as would be expected if rate limiting bacterial biosynthetic resources are diverted for virulence factor expression until a critical nutrient(s) is exhausted from the medium.

Protein cost is a major driving force in the shaping of regulatory systems (Dekel and Alon, 2005; Babu and Aravind, 2006; Kalisky *et al*., 2007; Stoebel *et al*., 2008; Gao and Stock, 2013). The rapid elimination of the prfA* genotype in the competition experiments in soil equates to a selection coefficient of about −0.33 d^−1^ (roughly a 33% difference in fitness measured over a day) (Lenski, 1992), indicating very strong selection against constitutive virulence gene expression in this environment. This selection is expected to be even greater in non‐sterile soil, where the presence of competing microbiota has been shown to significantly impair *L. monocytogenes* growth/survival (McLaughlin *et al*., 2011; Locatelli *et al*., 2013; Vivant *et al*., 2013). Whether expressing PrfA^WT^ or lacking the PrfA regulator, no significant differences in *L. monocytogenes* fitness were observed in either rich medium or soil. The cost neutrality of PrfA^WT^ in the tested extracellular conditions therefore indicates that the acquisition of an ON–OFF switchable PrfA regulator has been critical in the evolution of *L. monocytogenes* as a facultative parasite.

The instability in soil (but not BHI or other conditions) of the chromosomally integrated pPL2 constructs indicates that PrfA^WT^, and indeed the empty complementation vector itself, imposed a burden. This implies that soil is a strongly selective environment for *L. monocytogenes* in which, despite PrfA‐dependent genes being downregulated (Piveteau *et al*., 2011), any leaky expression due to the basal activity of PrfA^WT^ in the OFF state (Deshayes *et al*., 2012) may be disadvantageous. Indeed, although not apparent in BHI, Δ*prfA* bacteria also exhibit some fitness advantage over *prfA* ^WT^ bacteria in certain circumstances (e.g. chemically defined medium; our unpublished observations). *Listeria monocytogenes* possesses other mechanisms in addition to ON–OFF PrfA switching to ensure that the PrfA regulon is effectively silenced outside the host. For example, an RNA thermoswitch prevents efficient *prfA* gene translation at environmental temperatures (≤ 30°C) (Johansson *et al*., 2002). Growth on cellobiose and other plant‐derived β‐glucosides, presumably abundant in the decaying vegetation‐rich soil habitat, also strongly represses PrfA regulated genes (Brehm *et al*., 1999). The existence of these redundant PrfA‐downregulating mechanisms is consistent with preventing any virulence‐related fitness loss being critically important for *L. monocytogenes* outside the host.

Since dispensable genes tend to be readily eliminated from bacterial genomes (Cooper *et al*., 2001; Mira *et al*., 2001), *L. monocytogenes* is expected to lose the ability to express the PrfA regulon – and indeed the PrfA regulon altogether – during its existence as a free‐living organism. This appears to have occurred during evolution and is the presumed mechanism that gave rise to the obligate saprophytic species of the genus, typified by *Listeria innocua* (Vazquez‐Boland *et al*., 2001a; Schmid *et al*., 2005; Hain *et al*., 2006). Some strains of *Listeria seeligeri*, another non‐pathogenic species, still possess a partially conserved PrfA regulon undergoing gene decay processes. (Vazquez‐Boland *et al*., 2001a; den Bakker *et al*., 2010). Similarly, spontaneous *prfA* disabling mutations are not uncommon among *L. monocytogenes* food isolates (Roche *et al*., 2005). This predicts a scenario of rapid decline and even extinction of the pathogenic *L. monocytogenes*, which is clearly not supported by this species' known widespread distribution and epidemiology (Vazquez‐Boland *et al*., 2001b; Freitag *et al*., 2009). Arguably, therefore, virulence must somehow confer an evolutionary advantage to *L. monocytogenes*. The maintenance of the PrfA regulon may be positively selected in the environmental habitat for a number of reasons. For example, PrfA‐regulated virulence factors may promote survival by helping *Listeria* to evade predation by soil bacterivorous protozoa (Greub and Raoult, 2004). The PrfA regulon may also facilitate the subclinical colonization of the intestinal tract of animal hosts and subsequent fecal‐oral enrichment of virulent *L. monocytogenes* bacteria in the environment (Vazquez‐Boland *et al*., 2001b).

While essential for within‐host microbial proliferation, virulence, if excessive, may also reduce the time the infected host remains viable and producing pathogen offspring for transmission to new hosts. Based on this tenet, evolutionary theory posits that pathogen fitness is optimized through a trade‐off between virulence and transmission (Anderson and May, 1981; Antia *et al*., 1994; Bull, 1994; Bull and Lauring, 2014). This assumption, however, is host‐centric and based on direct host‐to‐host transmission models, neglecting that pathogens are also indirectly transmitted from environmental sources (Anderson and May, 1981; Roche *et al*., 2011; Mikonranta *et al*., 2012). Moreover, many pathogens, like *L. monocytogenes*, not only ‘sit‐and‐wait’ in the environment for new hosts (Walther and Ewald, 2004) but reproduce as free‐living organisms (Merikanto *et al*., 2012). Here, we provide with the facultative pathogen *L. monocytogenes* the first formal demonstration that virulence traits are intrinsically costly to the microbe, impairing pathogen proliferation outside the host. A significant implication is that, contrary to current belief (Bonhoeffer *et al*., 1996; Gandon, 1998; Walther and Ewald, 2004; Roche *et al*., 2011), the evolutionary dynamics of facultative pathogens that do not depend directly on a host for transmission is also constrained by a virulence‐transmission trade‐off. We suggest that this trade‐off has been a key determinant in the evolution of virulence regulation systems in facultative pathogens, as exemplified here with the *Listeria* PrfA switch. A deeper insight into how microbes control the costs of virulence both within and outside the host, and incorporating this knowledge into virulence theory, will be key to improve our understanding of pathogen ecology and the evolution of virulence.

## Experimental procedures

### Bacteria, plasmids, media and reagents

The strains and plasmids used are listed in Table 1. *Listeria monocytogenes* bacteria were all derived from the serovar 4b human isolate P14 (Ripio *et al*., 1996; 1997). *Listeria* and *Escherichia coli* were grown at 37°C in BHI (Difco‐BD) and Luria–Bertani (Sigma) media, respectively, supplemented with 1.5% agar (w/v) and/or antibiotics as appropriate. Chemicals and oligonucleotides were purchased from Sigma‐Aldrich unless stated otherwise.

**Table 1:** Bacterial strains and plasmids used in this study

Strain/plasmid	Genotype/description	Source (reference)	Internal strainq collection no.
*L. monocytogenes*			
P14	*prfA* ^WT^, wild‐type strain of serovar 4b, human clinical isolate	Our laboratory (Ripio *et al*., 1996; 1997)	PAM 14
P14A	prfA* ^G145S^ isogenic derivative of P14	Our laboratory (Ripio *et al*., 1996; 1997)	PAM 50
P14^REV^	*prfA* ^WT^, allele exchange wild‐type revertant of P14A	This study	PAM 3757
Δ*prfA*	In frame *prfA* deletion mutant of P14A	Our laboratory (Deshayes *et al*., 2012)	PAM 373
Δ*prfA* (vector)	Δ*prfA*, PAM 373 complemented with pPL2 empty vector	Our laboratory (Deshayes *et al*., 2012)	PAM 3293
Δ*prfA* (*prfA* ^WT^)	*prfA* ^WT^, PAM 373 complemented with pPL2prfAbc^WT^	This study	PAM 3319
Δ*prfA* (prfA*)	prfA* ^G145S^, PAM 373 complemented with pPL2prfAbc*	This study	PAM 3320
ΔREG	ΔLIPI‐1Δ*inlABΔinlCΔhpt*, PrfA regulon deletion mutant of P14A	This study	PAM 3691
ΔREG (vector)	PAM 3691 complemented with pPL2 empty vector	This study	PAM 3734
ΔREG (*prfA* ^WT^)	PAM 3691 complemented with pPL2prfAbc^WT^	This study	PAM 3694
ΔREG (prfA*)	PAM 3691 complemented with pPL2prfAbc*	This study	PAM 3695
ΔLIPI‐1	Δ*prfA plcA hly mpl actA plcB*, LIPI‐1 deletion mutant of P14A	This study	PAM 3732
ΔLIPI‐1 (vector)	PAM 3732 complemented with pPL2 empty vector	This study	PAM 3750
ΔLIPI‐1 (*prfA* ^WT^)	PAM 3732 complemented with pPL2prfAbc^WT^	This study	PAM 3751
ΔLIPI‐1 (prfA*)	PAM 3732 complemented with pPL2prfAbc*	This study	PAM 3752
Δ*inlABC*	Δ*inlAB*Δ*inlC* in frame deletion mutant of P14A	Our laboratory (unpublished)	PAM 3657
Δ*hpt*	Δ*hpt* in frame deletion mutant of P14A	Our laboratory (Scortti *et al*., 2006)	PAM 377
Δ*hly*	Δ*hly* in frame deletion mutant of P14A	Our laboratory (Deshayes *et al*., 2012)	PAM 3730
Δ*actA*	Δ*actA* in frame deletion mutant of P14A	Our laboratory (Suarez *et al*., 2001)	PAM 185
*E. coli*			
DH5α	Cloning host strain	Our laboratory	
Plasmids			
pPL2	Integrative vector for single‐copy gene complementation in *L. monocytogenes*	M. Loessner (Lauer *et al*., 2002)	
pMAD	Thermosensitive shuttle vector for allelic exchange in Gram‐positives	M. Debarbouille (Arnaud *et al*., 2004)	
pLSV1	Thermosensitive shuttle vector for allelic exchange in Gram‐positives	J. Kreft (Wuenscher *et al*., 1991)	
pPL2prfAbc^WT^	pPL2 inserted with PrfA‐autoregulated Δ*plcA*‐*prfA* ^WT^ bicistronic construct	This study	
pPL2prfAbc*	pPL2 inserted with PrfA‐autoregulated Δ*plcA*‐prfA*^G145S^ bicistronic construct	This study	
pLS5′ΔprfA^WT^	pLSV1 inserted with a 5′‐truncated *prfA* ^WT^ used in P14^Rev^ construction	This study	
pMΔLIPI‐1	pMAD inserted with recombinogenic construct for deletion of LIPI‐1	This study	
pLSVΔhpt	pLSV1 inserted with recombinogenic construct for deletion of *hpt*	Our laboratory	

### General DNA techniques

Chromosomal *Listeria* DNA was extracted and purified as previously described (Ripio *et al*., 1997). Plasmid DNA was extracted from *E. coli* using the Spin Miniprep kit from Qiagen and introduced into *L. monocytogenes* by electroporation (Ripio *et al*., 1997) using a Gene Pulser Xcell apparatus (Bio‐Rad). Polymerase chain reaction was carried out with Taq DNA polymerase (Biotools, Spain) for detection/mapping purposes or high‐fidelity ProofStart DNA polymerase (Qiagen) for mutant construction or gene complementation. The PCR products were purified with the PCR purification kit from Qiagen and analysed by standard gel electrophoresis in 1.0% agarose (Biotools). DNA sequences were determined on both strands by Sanger sequencing. Restriction enzymes were used according to the manufacturer's instructions (New England Biolabs).

### *prf* *A* ^WT^ revertant from *prf* *A* *

P14^Rev^ was constructed by replacing the prfA*^G145S^ allele of strain P14A with *prfA* ^WT^ following a procedure described in detail elsewhere (J. Monzó i Gil, PhD thesis, University of Bristol, UK, 2007). Briefly, primers PrfAalleI and PrfAalleII‐long (Table S1), the latter with a SalI site, were used to amplify the *prfA* gene from wild‐type *L. monocytogenes* P14 (Table 1). The PCR product was digested with SalI and EcoRI (naturally occurring internal site 25 bp downstream from the *prfA* start codon), and the resulting 5′ end‐truncated *prfA* fragment (which includes codon 145) was inserted into the thermosensitive shuttle vector pLSV1 (Wuenscher *et al*., 1991), giving rise to the allele replacement plasmid pLS5'ΔprfA (Table 1). After electroporation into P14A, integration of pLS5'ΔprfA^WT^ by homologous recombination was selected at 42°C in BHI supplemented with 5 μg ml^−1^ erythromycin. A single cross‐over recombinant colony was subcultured at 37°C in BHI without erythromycin in the presence of 7.5 μg ml^−1^ fosfomycin (disodium salt) to counterselect for reconstitution of the original prfA* allele of P14A in the second cross‐over event. This is possible thanks to the strictly PrfA‐dependent gene *hpt* encoding the organophosphate permease Hpt, which mediates uptake of (and hence susceptibility to) fosfomycin in *L. monocytogenes* (minimal inhibitory concentration > 256–512 μg ml^−1^ for *prfA* ^WT^, 2 μg ml^−1^ for prfA*) (Scortti *et al*., 2006). The *prfA* ^WT^ genotype of P14^Rev^ was confirmed by DNA sequencing. P14^Rev^ exhibited the characteristic PrfA phenotype of wild‐type *L. monocytogenes* as determined by PrfA functional assays (see below and Fig. S4).

### Deletion mutants and *prf* *A* complementation

Unmarked gene deletion mutants were constructed in *L. monocytogenes* P14A (Table 1) by allelic exchange using a thermosensitive shuttle vector. The in‐frame deletion mutants Δ*prfA*, Δ*hly*, Δ*actA*, Δ*hpt* and Δ*inlABC* were previously available in our laboratory (Table 1). For deleting LIPI‐1, DNA fragments of 893 bp and 684 bp corresponding to the chromosomal regions encompassing the *prfA* and *plcB* genes at each side of the pathogenicity island (see Fig. 1) were PCR‐amplified using primer pairs PrsF1/PrsR2 and PrsF3/PrsR4 (Table S1), then fused together by splicing overlap extension PCR (Pogulis *et al*., 1996) using the complementary 3′ sequence tails carried by PrsR2 and PrsF3 and a second PCR reaction with PrsF1 and PrsR4. The EcoRI and BamHI sites carried by the latter primers (Table S1) were used to insert the resulting 1577 bp PCR product into the pMAD vector (Arnaud *et al*., 2004), giving rise to the plasmid pMΔLIPI‐1 (Table 1). The ΔREG mutant was constructed by deleting LIPI‐1 and *hpt* from P14A Δ*inlABC* (Table 1). The *hpt* gene was in frame deleted using the pLSV1‐based pLSΔhpt allele replacement plasmid (Table 1). After electroporation, the first and second recombinants were selected and checked by PCR mapping as previously described (Suarez *et al*., 2001).

For *prfA* complementation, *prfA* ^WT^ and prfA*^G145S^ from P14 and P14A, respectively, with all native promoters including the PrfA‐dependent *plcA* promoter that positively autoregulates *prfA* expression (Mengaud *et al*., 1991; Scortti *et al*., 2007) (see Fig. 1), were inserted in monocopy in the *L. monocytogenes* chromosome using the integrative vector pPL2 (Lauer *et al*., 2002) as previously described (Deshayes *et al*., 2012). *prfA* constructs were generated by in‐frame deleting the *plcA* gene from the *plcA‐prfA* bicistron from either P14 or P14A by splicing overlap extension PCR using suitable primer combinations (Table S1). After electroporation into Δ*prfA* or ΔREG, pPL2 integrants were selected in BHI plates containing 7.5 μg ml^−1^ chloramphenicol. All gene deletions were confirmed by PCR and DNA sequencing.

### Western immunoblotting

*Listeria* were grown in 10 ml BHI until OD_600_ ≈ 1.0–1.2 and the cultures (1 ml) were centrifuged at ∼ 7000 × *g* for 5 min at 4°C to separate the supernatant and the bacterial cells. The cell‐free supernatant was precipitated with 16% trichloroacetic acid overnight at 4°C. After centrifugation at 18 000 × *g* for 10 min at 4°C, the protein pellet was washed with acetone, dried, then re‐suspended in 2% SDS 6 M urea Tris‐HCl buffer and stored at −80°C. For cell‐associated proteins, the bacterial pellet was re‐suspended in cold lysis solution (50 mM NaH_2_PO_4_, 300 mM NaCl, pH 7.4) with protease inhibitor cocktail (Roche), transferred to Lysis Matrix B tubes containing 0.1 mm silica beads (Q‐Biogene) and homogenized in a FastPrep instrument (Q‐Biogene) (three cycles of 30 s at speed set to 6). Cell debris was removed by centrifugation at 12 000 × *g* for 20 min at 4°C and the supernatant stored at −80°C. After determining total protein concentration (colorimetric DC protein assay, Bio‐Rad), protein samples were separated by SDS‐PAGE using 4–12% NuPAGE Bis–Tris mini gels (Novex Life Technologies) and electro‐transferred to polyvinylidene difluoride membranes using a Mini‐Protean II cuvette. Membranes were blocked for 2 h with 0.05% Tween 20 5% skim milk (w/v) phosphate‐buffered saline pH 7.2 (PBS) and incubated (1 h or overnight at room temperature) with appropriate primary (see below) and secondary (1:5000‐diluted anti‐rabbit and 1:2000‐diluted anti‐mouse, horseradish peroxidase‐conjugated) antibodies in the same solution. After washing, immunoreactive proteins were detected using Amersham's ECL chemiluminescent detection reagents (GE Healthcare). The following primary antibodies were used: PrfA rabbit polyclonal (Vega *et al*. 1998); PlcA and PlcB mouse monoclonals (J. Wehland, Braunschweig, Germany); Hly mouse monoclonal (T. Chakraborty, Giessen, Germany); InlA and InlB mouse monoclonals (P. Cossart, Paris, France); and InlC rabbit polyclonal (raised against an InlC‐specific peptide).

### Growth curves

Overnight BHI cultures were diluted 1:100 into fresh BHI and grown at 37°C with rotary shaking (200 r.p.m.) until ≈ 1.0 OD_600_. Bacteria were collected by centrifugation, washed twice in PBS and suspended in pre‐warmed BHI to give an OD_600_ = 0.05. Triplicate 200 μl aliquots of the bacterial suspensions were transferred to different positions of flat‐bottom 96‐well microplates (Costar). Plates were incubated at 37°C with shaking (200 r.p.m.) and bacterial growth monitored by measuring the OD_600_ every 30 min in an automated plate reader (FluoStar Optima or Omega machines, BMG Labtech). Cultures were monitored by phase‐contrast microscopy to exclude bacterial clumping as a potential source of variation. The maximum growth rate during exponential growth (μ) and maximum bacterial cell density reached during the growth curve (A) were estimated from spline‐fits of OD_600_ values using the grofit package in R (Kahm *et al*., 2010).

### Intracellular infection assay

*Listeria monocytogenes* intracellular proliferation was tested in human epithelial HeLa cell monolayers using a gentamicin protection assay as previously described (Deshayes *et al*., 2012). Due to the constitutive activation of their PrfA‐regulated cell invasion determinants, prfA* bacteria are more invasive than (broth‐grown) *prfA* ^WT^ bacteria (see Fig. 3, upper panel). Intracellular proliferation data were therefore normalized to the number of internalized *L. monocytogenes* bacteria using an intracellular growth coefficient calculated with the formula: IGC = (IB_n_ − IB_0_) / IB_0_, where IB_n_ and IB_0_ are the intracellular bacterial numbers at any specific time point (*t* = n) and *t* = 0, respectively (Deshayes *et al*., 2012).

### Soil experiments

For each experiment, subsurface topsoil samples were collected within a depth of ≈ 10 cm from several locations of a residential garden in Edinburgh (UK). Soil was carefully mixed, sieved through 6 mm mesh to remove coarse particles and autoclaved (121°C‐15 min). The soil used had a pH of 7.23 (range 7.2–7.3) and average moisture content of 25.3% (range 24.1 and 26.5). The pH was measured in the liquid phase of a soil suspension prepared by vigorously stirring 25 g of soil in 50 ml distilled water. The water content was determined in 10 g samples by the oven‐dry method. Prior to the experiments, the soil was tested for the presence of antimicrobial or inhibitory activity against *L. monocytogenes* (P14A, P14^Rev^ and Δ*prfA*). For this, a soluble extract was prepared by suspending 50 g of soil in 50 ml distilled water. After mixing vigorously, the suspension was left to sediment for 20 min at room temperature and the supernatant filtered through 0.22 μ pore size membranes. No inhibition zones were observed in lawn cultures when drops of the soil filtrate were applied onto BHI plates seeded with the three test strains. Growth inhibition assays in fluid BHI culture also failed to detect inhibitory activity in the soil filtrate. For growth assays, sterile soil (≈ 450 g per experiment) was inoculated with (≈ 45 ml) twice‐washed *Listeria* cell suspensions in PBS and thoroughly homogenized for 5 min in a blender. Bacterial inocula were prepared from exponential BHI cultures as above indicated. Random samples were taken to confirm the uniform distribution of the inoculum. Microcosms (three per time point) were prepared by aseptically transferring ≈ 45 g of inoculated soil into Falcon tubes and incubated at room temperature in static conditions, without exposure to sunlight and at constant moisture. At the specified time points, two 1‐g soil samples per replicate were vigorously vortexed for 20 s with 1.5 ml diluent (PBS containing 0.05% trypsin and 0.9 mM 4Na 2H_2_O EDTA to ensure optimal bacterial recovery) in 15 ml Falcon tubes, the suspension allowed to settle for 5 min, and the supernatant decimally diluted and plated for viable count determination. The relative frequencies of the competing strains were determined by analysing at least 50 randomly selected colonies by PrfA phenotyping (see below) and PCR using primers PrfAalleI and PrfAalleII‐long (Table S1) for detection of the Δ*prfA* deletion. The log cfu numbers for each strain inferred from their frequency data were used to calculate their competitive index using the formula CI = (test/reference log cfu ratio at *t* = n)/(test/reference log cfu ratio at *t* = 0).

### Strain characterization

The *prfA* genotype of the strains was confirmed by DNA sequencing and the corresponding phenotypes systematically checked using PrfA functional assays. The latter are based on a panel of tests that detect the activity of the products of specific PrfA‐regulated genes used as natural reporters of PrfA activation status, namely: haemolysin activity (*hly* gene) in sheep blood agar (Biomérieux) (Fig. S4, left panel); phospholipase activity (*plcB* gene) in egg yolk BHI agar (Ripio *et al*., 1996; Vega *et al*., 2004) (Fig. S4, centre panel); and fosfomycin susceptibility (*hpt* gene) (Scortti *et al*., 2006). Phospholipase and fosfomycin susceptibility was also tested in charcoal (0.5% w/v)‐supplemented BHI plates (BHIC) to determine PrfA^WT^ activability (Ermolaeva *et al*., 2004; Scortti *et al*., 2006). Activated charcoal sequesters a diffusible PrfA repressor from the culture medium, leading to partial activation of PrfA‐dependent gene expression (Ermolaeva *et al*., 2004) (see Fig. S4, right panel). Using these tests, *L. monocytogenes prfA* ^WT^ is characterized by (i) weak haemolysis (confined to area underneath the colonies), (ii) no PlcB activity and resistance to fosfomycin in BHI, and (iii) strong PlcB activity and susceptibility to fosfomycin in BHIC. prfA* bacteria, in contrast, exhibit (i) strong haemolysis (wide halo extending beyond the colonies), (ii) strong PlcB activity and fosfomycin susceptibility in BHI, and (iii) equally strong PlcB activity and fosfomycin susceptibility in BHIC. Δ*prfA* bacteria are phenotypically distinguishable from *prfA* ^WT^ bacteria since the former remain PlcB negative and resistant to fosfomycin in BHIC.

### Statistics

Growth parameters were analysed using one‐way ANOVA followed by Šidák post‐hoc multiple comparison tests unless otherwise stated. Two‐way ANOVA was used to compare intracellular proliferation data. One‐sample Student's *t*‐tests were used to determine if CI values differed significantly from 1 (the theoretical CI value if the ratio of the competing strains remains the same respect to *t* = 0). Prism 6.0 (GraphPad, San Diego, CA) or Minitab 16 (Minitab, State College, PA) statistical software was used.

## Supporting information


**Fig. S1.** Growth of Δ*inlABC* and Δ*hpt* compared with their parent prfA* strain P14A and isogenic *prfA* ^WT^ (P14^Rev^) and Δ*prfA* P14A derivatives in BHI. Mean ± SEM of four experiments. (A) Growth curves. (B) Corresponding μ (growth rate) and A (maximum growth) values. prfA* strain P14A used as reference in post‐hoc multiple comparison. Numbers indicate *P* values; ns, not significant.
**Fig. S2.** Growth of in frame Δ*hly* mutant compared with its parent prfA* strain P14A and isogenic *prfA* ^WT^ (P14^Rev^) and Δ*prfA* in BHI. Mean ± SEM of at least three experiments. (A) Growth curves. (B) Corresponding μ (exponential growth rate) and A (maximum growth) values. prfA* strain P14A used as reference in post‐hoc multiple comparison. Numbers indicate *P* values; ns, not significant.
**Fig. S3.** Growth of in frame Δ*actA* mutant compared with its parent prfA* strain P14A and isogenic *prfA* ^WT^ (P14^Rev^) and Δ*prfA* in BHI. Mean ± SEM of at least three experiments. (A) Growth curves. (B) Corresponding μ (exponential growth rate) and A (maximum growth) values. prfA* strain P14A used as reference in post‐hoc multiple comparison. Numbers indicate *P* values; ns, not significant.
**Fig. S4.** PrfA phenotype testing. Typical phenotypes of prfA* (P14A), *prfA* ^WT^ (P14^Rev^) and Δ*prfA* bacteria on sheep blood agar (left), egg yolk‐BHI agar (centre) and egg yolk‐BHI agar supplemented with 0.5% (w/v) activated charcoal (right). Note in *L. monocytogenes prfA* ^WT^ the typical activation of PrfA‐dependent expression in charcoal‐supplemented medium as revealed using the activity of the *plcB* gene (PlcB phospholipase) as a reporter (indicated by black triangle). See *Experimental procedures* for details.
**Fig. S5.** Stability of PrfA phenotypes from P14A (prfA*) and P14^Rev^ (*prfA* ^WT^) strains in soil. The PrfA phenotype of soil isolates was systematically checked using a battery of functional tests (see Experimental *procedures* and Fig. S4). Example shown corresponds to haemolysin phenotype screening on sheep blood agar of *L. monocytogenes* P14A and P14^Rev^ colonies from the experiment in Fig. 7. Controls: streaks of the originally inoculated (1) P14A, (2) P14^Rev^ and (3) Δ*prfA* bacteria.
**Table S1.** Main oligonucleotides used in this study. Relevant restriction sites are underlined; overlapping sequences for recombinant PCR are in lower case.
