# Supplementary material for: PrfA regulation offsets the cost of L isteria virulence outside the host
Source: Environ Microbiol. 2015 Aug 27;17(11):4566–79. doi: 10.1111/1462-2920.12980 (PMC4737189; doi:10.1111/1462-2920.12980)
Supplement: Supplementary file 1 — Fig. S1. Growth of ΔinlABC and Δhpt compared with their parent prfA* strain P14A and isogenic prfA WT (P14Rev) and ΔprfA P14A derivatives in BHI. Mean ± SEM of four experiments. (A) Growth curves. (B) Corresponding μ (growth rate) and A (maximum growth) values. prfA* strain P14A used as reference in post‐hoc multiple comparison. Numbers indicate P values; ns, not significant. Fig. S2. Growth of in frame Δhly mutant compared with its parent prfA* strain P14A and isogenic prfA WT (P14Rev) and ΔprfA in BHI. Mean ± SEM of at least three experiments. (A) Growth curves. (B) Corresponding μ (exponential growth rate) and A (maximum growth) values. prfA* strain P14A used as reference in post‐hoc multiple comparison. Numbers indicate P values; ns, not significant. Fig. S3. Growth of in frame ΔactA mutant compared with its parent prfA* strain P14A and isogenic prfA WT (P14Rev) and ΔprfA in BHI. Mean ± SEM of at least three experiments. (A) Growth curves. (B) Corresponding μ (exponential growth rate) and A (maximum growth) values. prfA* strain P14A used as reference in post‐hoc multiple comparison. Numbers indicate P values; ns, not significant. Fig. S4. PrfA phenotype testing. Typical phenotypes of prfA* (P14A), prfA WT (P14Rev) and ΔprfA bacteria on sheep blood agar (left), egg yolk‐BHI agar (centre) and egg yolk‐BHI agar supplemented with 0.5% (w/v) activated charcoal (right). Note in L. monocytogenes prfA WT the typical activation of PrfA‐dependent expression in charcoal‐supplemented medium as revealed using the activity of the plcB gene (PlcB phospholipase) as a reporter (indicated by black triangle). See Experimental procedures for details. Fig. S5. Stability of PrfA phenotypes from P14A (prfA*) and P14Rev (prfA WT) strains in soil. The PrfA phenotype of soil isolates was systematically checked using a battery of functional tests (see Experimental procedures and Fig. S4). Example shown corresponds to haemolysin phenotype screening on sheep blood agar of L. monocytogenes P [file EMI-17-4566-s001.pdf]

SUPPORTING INFORMATION

Supplementary Figures

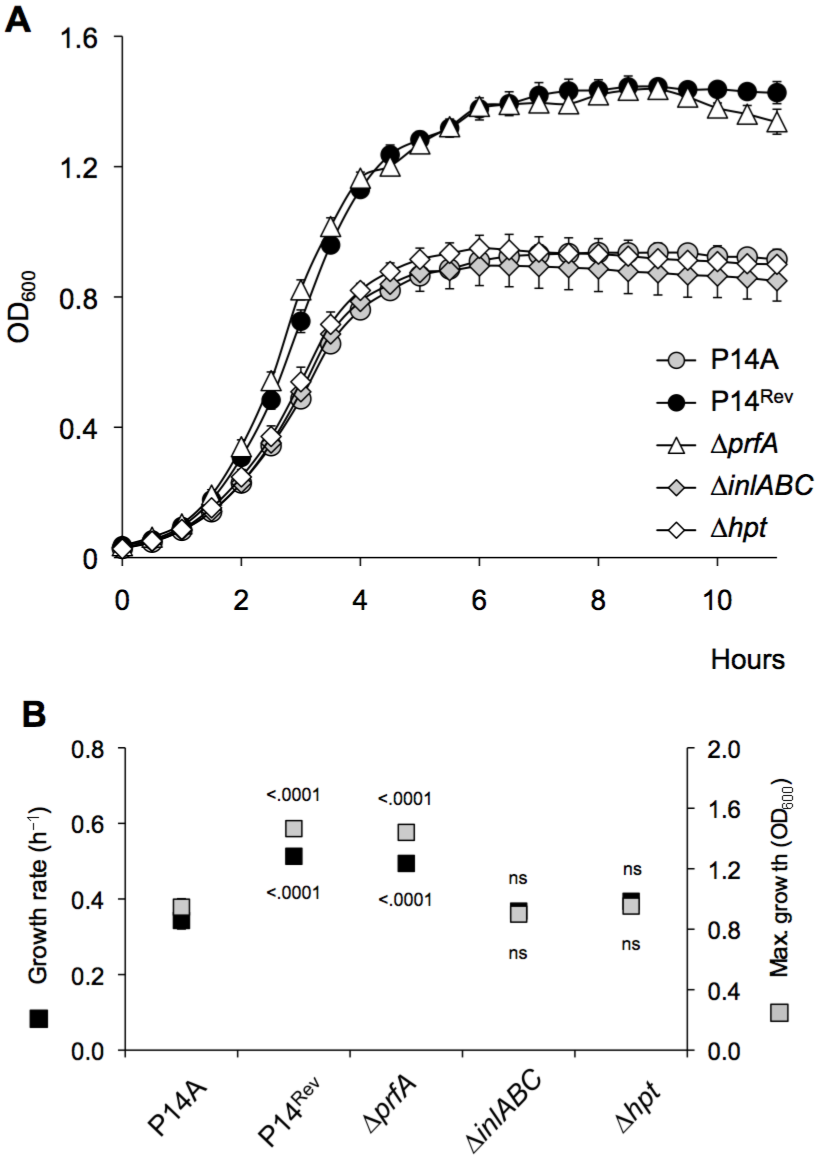

Figure S1. Growth of Δ*inlABC* and Δ*hpt* compared to their parent *prfA** strain P14A and isogenic *prfA*^{WT} (P14^{Rev}) and Δ*prfA* P14A derivatives in BHI. Mean ±SEM of four experiments. (A) Growth curves. (B) Corresponding μ (growth rate) and A (maximum growth) values. *prfA** strain P14A used as reference in post-hoc multiple comparison. Numbers indicate *P* values; ns, not significant.

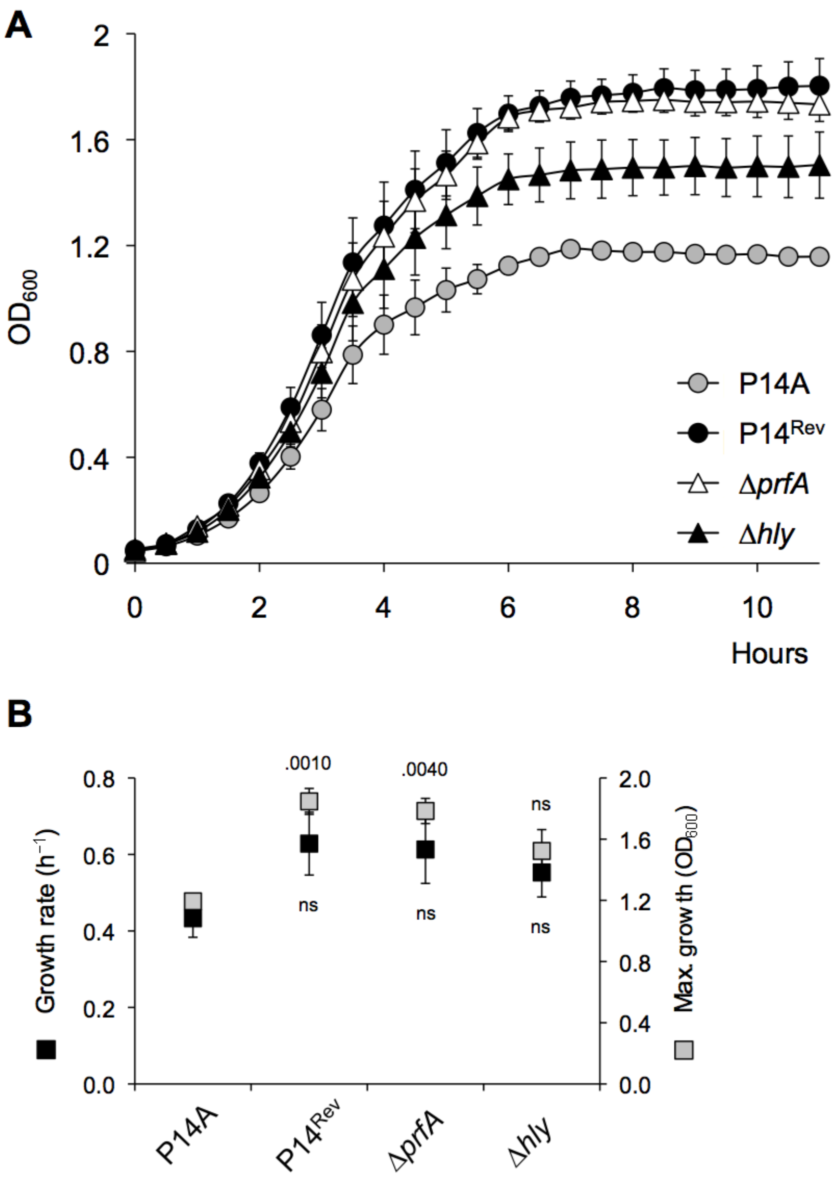

Figure S2. Growth of in-frame Δ*hly* mutant compared to its parent *prfA** strain P14A and isogenic *prfA*^{WT} (P14^{Rev}) and Δ*prfA* in BHI. Mean ±SEM of at least three experiments. (A) Growth curves. (B) Corresponding μ (exponential growth rate) and A (maximum growth) values. *prfA** strain P14A used as reference in post-hoc multiple comparison. Numbers indicate *P* values; ns, not significant.

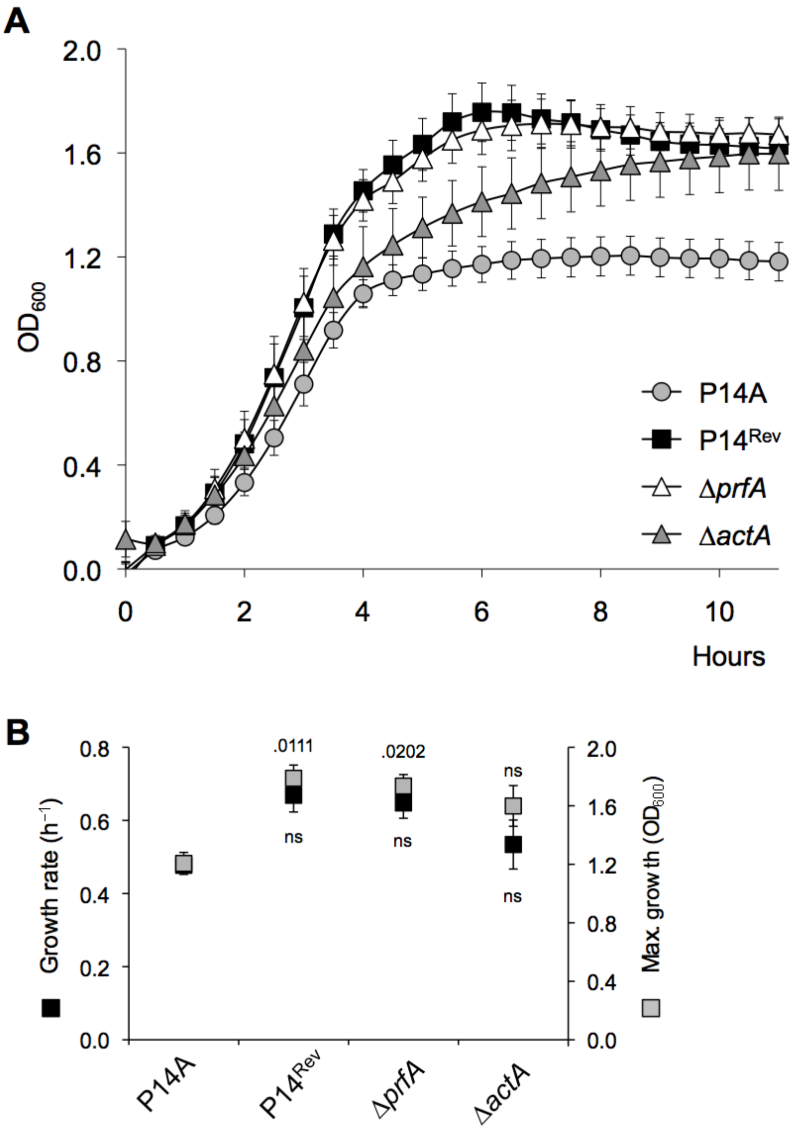

Figure S3. Growth of in-frame Δ*actA* mutant compared to its parent *prfA** strain P14A and isogenic *prfA*^{WT} (P14^{Rev}) and Δ*prfA* in BHI. Mean ±SEM of at least three experiments. (A) Growth curves. (B) Corresponding μ (exponential growth rate) and A (maximum growth) values. *prfA** strain P14A used as reference in post-hoc multiple comparison. Numbers indicate *P* values; ns, not significant.

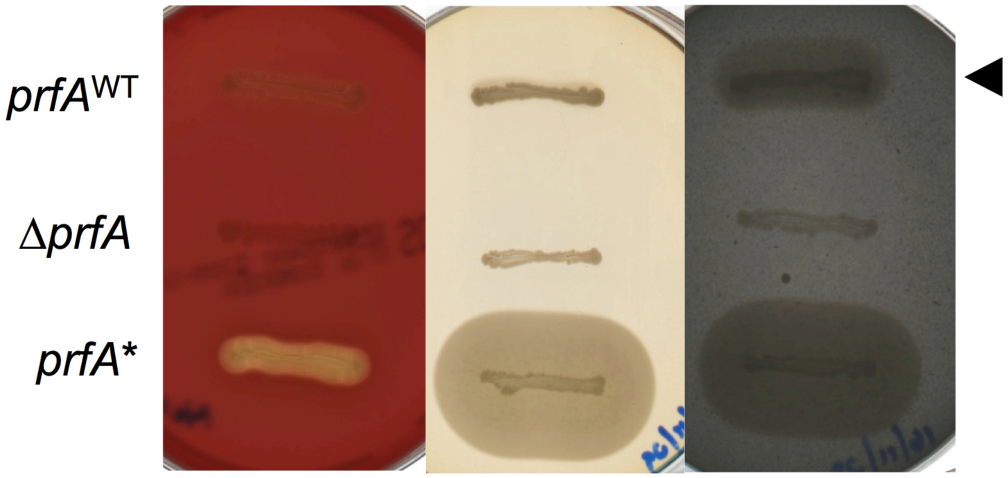

Figure S4. PrfA phenotype testing. Typical phenotypes of *prfA** (P14A), *prfA*^{WT} (P14^{Rev}) and Δ*prfA* bacteria on sheep blood agar (left), egg yolk-BHI agar (centre) and egg yolk-BHI agar supplemented with 0.5% (w/v) activated charcoal (right). Note in *L. monocytogenes prfA*^{WT} the typical activation of PrfA-dependent expression in charcoal-supplemented medium as revealed using the activity of the *plcB* gene (PlcB phospholipase) as a reporter (indicated by black triangle). See Experimental Procedures for details.

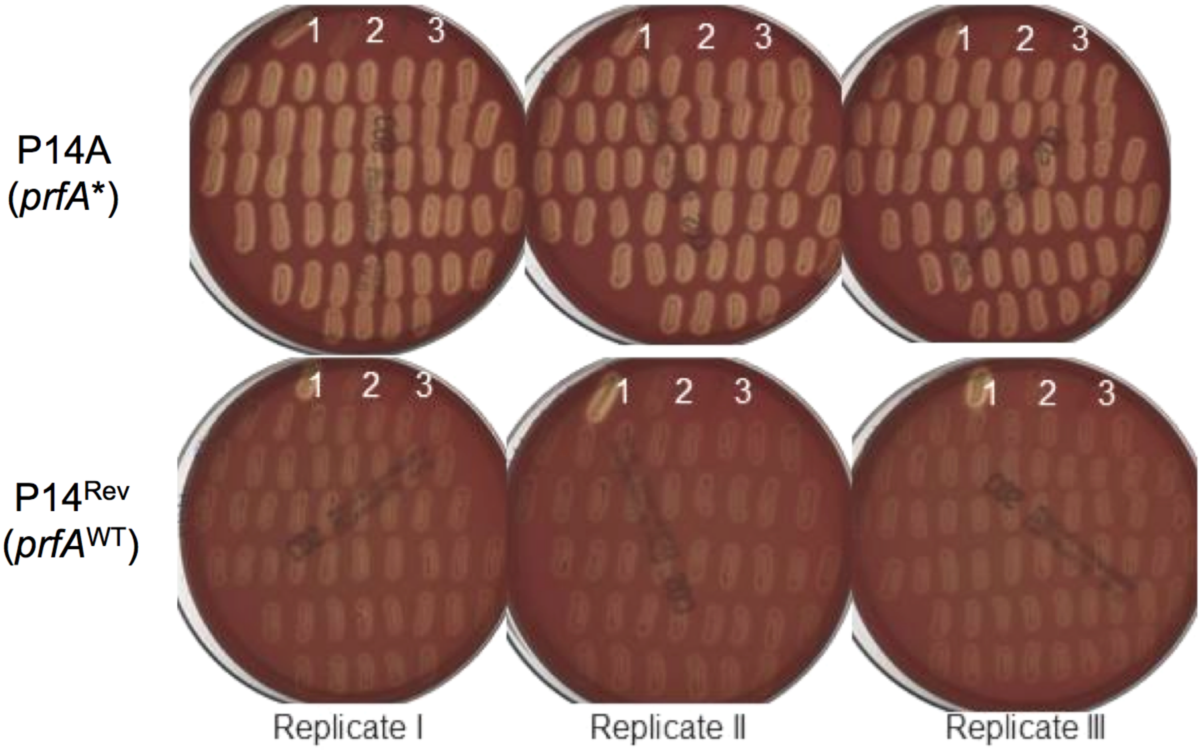

Figure S5. Stability of PrfA phenotypes from P14A (*prfA**) and P14^{Rev} (*prfA*^{WT}) strains in soil. The PrfA phenotype of soil isolates was systematically checked using a battery of functional tests (see Experimental Procedures and Fig. S4). Example shown corresponds to hemolysin phenotype screening on sheep blood agar of *L. monocytogenes* P14A and P14^{Rev} colonies from the experiment in Fig. 7. Controls: streaks of the originally inoculated (1) P14A, (2) P14^{Rev} and (3) Δ*prfA* bacteria.

Table S1. Main oligonucleotides used in this study. Relevant restriction sites are underlined; overlapping sequences for recombinant PCR are in lower case.

Primer	Sequence 5'→3'	Use
PrsF1	CGCGAATTCGAAGAAAGTATCCGTGGTTGTC	ΔLIPI-1 deletion
PrsR2	TTTGCTGTGAATCTCTTAGTCTACTTGGGGAAAATTAAATTAAATC	
PrsF3	GATTTAATTTAATTTTCCCCAAGTAGACTAAGAGATTCACAGCAAA	
PrsR4	CGCGGATCCCGACTAACATAACCGCCATAATC	
Primer5	CATTTACATGGGAGATGCGGAAATTG	Checking of ΔLIPI-1 deletion
Primer6	CGAAATGGAACAACGTGCGAAAG	
PrfAalleI	CAGATAACAATTGTTGTTACTGCC	PCR Amplification of *prfA* gene for screening, DNA sequencing, preparation of pLS5'ΔprfA^{WT}
PrfAalleII-long	GATGCGTCGACGAGACATCCTGTTTT	
MMR9*Kpn*IR9*Kpn*I	CTAGGGTACCTTCGCTTCTAAAGATGAAACG	Construction of pPL2prfAbc$^{WT/*}$ constructs for *prfA* complementation
delplcALINKanti	atatatgttagttgaaATAATTCTTATACAAA	
MR10*Spe*I	CTAGACTAGTCTTGGTGAAGCAATCGTACGC	
delplcALINK	TTTGTATAAGAATTATttcaactaacatatat	
